# How should teaching on whole person medicine, including spiritual issues, be delivered in the undergraduate medical curriculum in the United Kingdom?

**DOI:** 10.1186/s12909-015-0378-2

**Published:** 2015-06-02

**Authors:** Mark T. Harbinson, David Bell

**Affiliations:** Centre for Medical Education, The Queen’s University of Belfast, 97 Lisburn Road, Belfast, BT9 7BL, Northern Ireland UK

## Abstract

**Background:**

Although the General Medical Council recommends that United Kingdom medical students are taught ‘whole person medicine’, spiritual care is variably recognised within the curriculum. Data on teaching delivery and attainment of learning outcomes is lacking. This study ascertained views of Faculty and students about spiritual care and how to teach and assess competence in delivering such care.

**Methods:**

A questionnaire comprising 28 questions exploring attitudes to whole person medicine, spirituality and illness, and training of healthcare staff in providing spiritual care was designed using a five-point Likert scale. Free text comments were studied by thematic analysis. The questionnaire was distributed to 1300 students and 106 Faculty at Queen’s University Belfast Medical School.

**Results:**

351 responses (54 staff, 287 students; 25 %) were obtained. >90 % agreed that whole person medicine included physical, psychological and social components; 60 % supported inclusion of a spiritual component within the definition. Most supported availability of spiritual interventions for patients, including access to chaplains (71 %), counsellors (62 %), or members of the patient’s faith community (59 %). 90 % felt that personal faith/spirituality was important to some patients and 60 % agreed that this influenced health. However 80 % felt that doctors should never/rarely share their own spiritual beliefs with patients and 67 % felt they should only do so when specifically invited. Most supported including training on provision of spiritual care within the curriculum; 40-50 % felt this should be optional and 40 % mandatory. Small group teaching was the favoured delivery method. 64 % felt that teaching should not be assessed, but among assessment methods, reflective portfolios were most favoured (30 %). Students tended to hold more polarised viewpoints but generally were more favourably disposed towards spiritual care than Faculty. Respecting patients’ values and beliefs and the need for guidance in provision of spiritual care were identified in the free-text comments.

**Conclusions:**

Students and Faculty generally recognise a spiritual dimension to health and support provision of spiritual care to appropriate patients. There is lack of consensus whether this should be delivered by doctors or left to others. Spiritual issues impacting patient management should be included in the curriculum; agreement is lacking about how to deliver and assess.

**Electronic supplementary material:**

The online version of this article (doi:10.1186/s12909-015-0378-2) contains supplementary material, which is available to authorized users.

## Background

Health has been defined by The World Health Organization (WHO) as a state of ‘complete physical, mental and social wellbeing and not merely the absence of disease or infirmity’ [1]. Subsequently, addition of a spiritual dimension to this definition has been advocated [2], reflected in recent quality of life questionnaires [3]. ‘Whole person medicine’ is a collective term used to describe all aspects of care required to restore and improve health in an individual, comprising physical, psychological, social, cultural and spiritual elements [4]. Psychological and social aspects of illness are frequently addressed during routine clinical assessment, while spiritual aspects are often neglected [5, 6]. Spirituality is defined as ‘personally held beliefs, values, and practices’ and ‘awareness of the ultimate meaning and purpose of life’ and may be associated with, but should be differentiated from, religion or religiosity, which imply ‘an expression of spiritual belief through an organized system of rituals and practices’ [7]. The therapeutic strategy linked to this domain of health has sometimes been called spiritual care, defined as ‘recognizing and responding to the multifaceted expressions of spirituality we encounter in our patients and their families. It involves compassion, presence, listening and the encouragement of realistic hope, and might not necessarily include any discussion of God or religion’ [8].

Given the high background level of religious belief in many Western countries, it is not unexpected that patients often view spiritual issues as important during illness and when making healthcare-related decisions [9] and want their carers, including healthcare professionals, to engage with them in addressing such matters [10, 11]. Conversely, in Silvestri’s study exploring factors influencing whether to undergo treatment for cancer [9], although clinicians, patients and caregivers agreed that the recommendation of an oncologist was the most important factor influencing the decision, patients and caregivers placed faith in God second, in contrast to physicians who ranked this as of less importance than effectiveness and side-effects of treatment, recommendation of family doctor and views of family members. Less than half of United States of America (USA) primary care residents felt that they should engage in spiritual care of their patients; the likelihood of viewing such interventions favourably was strongly linked to the physician’s own spirituality and to severity of the patient’s illness [12]. Fewer studies have addressed the views of medical students, with conflicting findings regarding the importance American medical students attribute to spiritual issues for patient management [13, 14]. Student attitudes towards spirituality in medicine may change with increasing exposure to such concepts as they progress through the longitudinal curriculum [15]. There have been limited studies conducted outside United States, where most entrants to medical school are almost exclusively older and graduates; training, experience and societal influences may differ from that in other countries.

The Scottish Executive Health Department has placed some responsibility on doctors, as members of the multidisciplinary team, in delivery of spiritual care tailored to the needs of the population [16]. General Medical Council of United Kingdom (GMC, UK) guidance ‘Tomorrow’s Doctors’ [17] also recommends that medical students appreciate ‘the importance of clinical, psychological, spiritual, religious, social and cultural factors’, and respect ‘patients’ right to hold religious or other beliefs’. The guidance however goes further than simple recognition of spiritual views as important to patients and their attitudes to illness, by stipulating that doctors should ‘ take these into account when relevant to treatment options’. Clearly there is an obligation to provide students with opportunities to avail of training themselves in this area.

Publications relating to incorporation of spirituality into medical education are almost exclusively related to studies conducted in USA [18] where the majority of medical schools have delivered teaching in spirituality and healthcare, in some cases for more than 20 years. There is little detailed published information on what specifically is covered, and considerable diversity regarding methods employed, with lectures, small group discussion, patient interviews, meetings with chaplains, and self-directed learning, predominantly reading, all reported [19, 20]. Often teaching on spirituality is immersed in an overarching theme encompassing ethics, sociology, diversity and related medical humanities [21–23]. Nor is there consensus as to what should be included, when the material should be delivered, by whom, and which methods should be employed for delivery and assessment. There is however generally a greater emphasis on clinical application, facilitated through small group teaching/discussion and case-based learning, rather than didactic teaching of theoretical concepts. Others have attempted to develop material for medical graduates: Anandarajah [24] identified a set of core competencies in spiritual care, encompassing knowledge, skills and attitudes, as a basis for developing a curriculum for use in American family practice although the authors noted that certain aspects may be adaptable to undergraduate medical education.

From the sparse data published [25, 26], amongst the minority of UK medical schools, including Queen’s University Belfast (QUB) [27], which address the GMC guidance on spirituality in healthcare in the undergraduate medical curriculum and seem currently to provide some training in spiritual issues, content and approach varies considerably and a diverse range of teaching and assessment methods prevail. Some schools do provide core teaching on the importance of faith in coping with illness for some patients, and information on how to liaise with chaplaincy services. However teaching is more frequently optional, delivered to small numbers within the student selected component programme or intercalated degree pathways, rather than the core undergraduate medical curriculum. Only four of the medical schools provided opportunities to accompany chaplains on patient visits. Few studies have reported the views of UK medical school teaching Faculty and students.

Both WHO and GMC recognise the importance of spirituality to health, and to the response to disease. Although GMC requires that UK medical schools address such issues in the undergraduate curriculum [17], the extent and content of such current teaching is not particularly clear. Surveys of practice at medical schools are dominated by data from USA, and recent review articles have called for studies from elsewhere in the world [18]. Our aim was to ascertain attitudes of medical students and their teachers towards whole-person medicine and, specifically, spirituality and its relationship to health and disease, and to determine their views on whether and, if so, how such issues should be addressed in the UK undergraduate medical curriculum, and what forms of teaching and assessment might be appropriate.

## Methods

### Study design

A self-administered questionnaire was devised to elicit views of medical students and of Faculty. An additional word document file reproduces the questionnaire in full [Additional file 1]. To enable standardization and facilitate data analysis, a five point Likert scale was chosen for each response.

The first part of the questionnaire gathered demographic information. Subsequent study questions were grouped into three domains, structured as follows:

- Domain 1: attitudes to whole person medicine

Respondents graded their attitudes to the various components of whole person care (physical, psychological, social, spiritual) on a scale ranging from irrelevant to very important.

- Domain 2: attitudes to spirituality and illness

This domain explored issues related to the interplay between spirituality and health, including the contribution of spiritual beliefs to health status. Respondents were asked to explore the relationship between patient and physician spirituality and whether medical staff should share personal views on spirituality with patients.

- Domain 3: attitudes to the training of healthcare staff in spiritual care

This domain focused on medical education and explored views on the training and assessment of medical students in spiritual aspects of healthcare.

The questionnaire ended with a free text box to allow any views expressed to be expanded or clarified, and to facilitate any other comments on the subject.

Following piloting of the questionnaire with a small group of students and Faculty, and advice from a medical statistician, the questionnaire was refined and ethical approval for use was granted by QUB Medical School’s Research Ethics Committee. The questionnaire was anonymous and no personal identifiable data were sought or stored; participation was entirely voluntary and (non)-participation had no repercussions for academic performance or career progression. All QUB undergraduate medical students willing to consent were eligible. Students were stratified by stage in their training into early (Years 1 and 2) and late (Years 3–5) groups. Similarly, all members of academic staff actively involved in undergraduate medical student education at QUB and willing to consent were eligible. Faculty were stratified by clinical and non-clinical background.

All potential participants were invited to participate via an email containing a hyperlink to the questionnaire, transcribed using SurveyMonkey software. Responses were automatically logged by the SurveyMonkey program. One reminder email was sent two weeks after the first invitation and the questionnaire closed for responses one week later.

### Statistical considerations

A minimum of 80 students in each cohort (Years 1–2, Years 3–5) was required to detect a 0.5 difference in a 5 point ordinal scale questionnaire with 90 % power. Approximately 250–270 students are enrolled in each year of the medical course at QUB. Given the generally low response rate from questionnaire surveys (typically, 30 %) and the difficulties in designing an alternative study method with appropriate stratified sampling, the invitation to participate was sent to all undergraduate medical students.

Quantitative (including comparison of proportions selecting the various options and relations between groups), and qualitative (assessing trends/themes) analysis was performed as appropriate. Simple descriptive statistics were used to describe the distribution of responses for each question. A Chi squared test for trend analysis was undertaken to compare responses between students and Faculty, and between Years 1–2 and Years 3–5 students, to investigate if increasing clinical exposure and experience leads to any differences in views. If any answer category contained fewer than 5 counts, it was summed with the adjacent cell. A statistically significant result was defined as *P* < 0.05. The free text comments were analysed by thematic analysis.

## Results

### Demographics of respondents

Characteristics of the study population are presented in Table 1. Overall 351 responses were obtained comprising Faculty (evenly distributed between clinical and non-clinical) and medical students. The majority of respondents were <25 years old with the remainder comprising graduate entrants and Faculty. ~60 % respondents were female and >90 % were from the British Isles. Typically there was a 25-30 % response from each student year group except Year 4 (10 %), most probably because many were on overseas clinical electives at the time the survey was undertaken. However, the Years 1–2 cohort combined accounted for 128 respondents, and Years 3–5 cohort 156 respondents which exceeded the target recruitment of 80 students per cohort, indicative that the study had >90 % power to detect a difference of 0.5 points on the ordinal scale for each question. There was a high return from amongst Faculty, exceeding 50 % of eligible participants.

**Table 1:** Demographic details of study subjects (*n* = 351 respondents)

Characteristics	Response percent	Response count
Respondent status		
QUB staff-non clinical	7.6 %	26
Medical student-school leaver	71.6 %	244
Medical student- graduate entry	12.6 %	43
Age		
</=20 years	19.2 %	65
21-25 years	58.6 %	198
26-30 years	6.2 %	21
>31 years	16.0 %	54
Gender		
Male	38.9 %	132
Female	61.1 %	207
Country of birth		
N. Ireland	77.3 %	262
Republic of Ireland	5.6 %	19
Great Britain	10.3 %	35
Europe	0.3 %	1
Rest of World	6.5 %	22
Year of study (medical student respondents)		
First	20.1 %	57
Second	25.0 %	71
Third	20.4 %	58
Fourth	10.2 %	29
Fifth	24.3 %	69

### Attitudes to whole person medicine

**Fig. 1:**
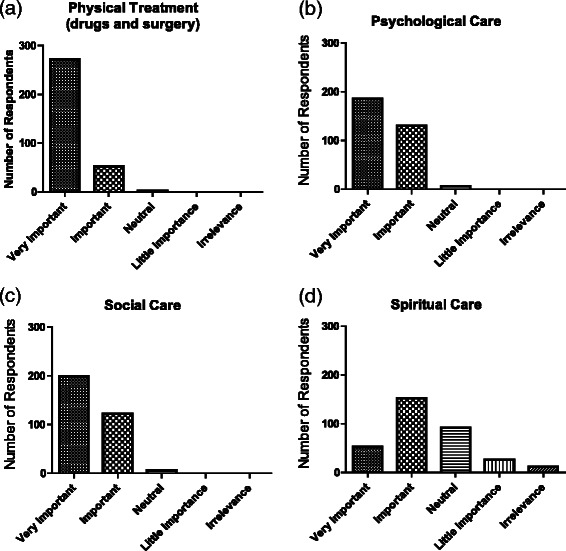
Attitudes of study respondents to the various components of whole person care, including **a** physical, **b** psychological, **c** social and **d** spiritual aspects, expressed as absolute numbers of respondents

**Fig. 2:**
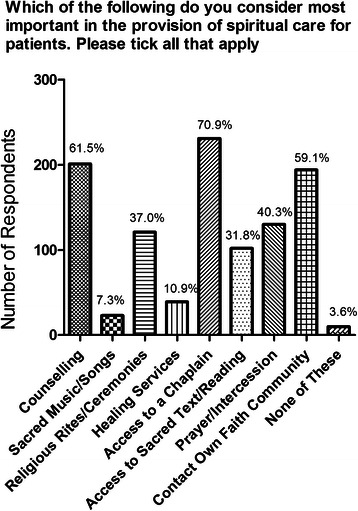
Attitudes to various components of spiritual care, expressed as absolute numbers (y axis) and as percentages (given on columns)

Attitudes to the various aspects of whole person care are shown in Fig. 1. Not surprisingly, almost all respondents indicated that physical treatment including the use of drugs or medical therapy was important or very important in patient management. A very high proportion also endorsed social care and psychological care as important or very important although the strength of feeling appeared somewhat less than for physical care with fewer respondents rating these aspects as ‘very’ important. Given the high scores accorded to each of areas involved in whole person care, it is not surprising that there was no difference in responses between Faculty and students, or between Years 1–2 and Years 3–5 students (data not shown). Overall, fewer respondents (61 %) rated spiritual care as important or very important in patient management, with the clear majority selecting important rather than very important (Fig. 1d). The data therefore appear to show a graded response with small reductions in the strength of support moving from physical through psychological and social care to spiritual care. Components of spiritual care for patients rated as most important by respondents were access to a chaplain, availability of counselling, and contact with a member of the patient’s own faith community (Fig. 2). There is some overlap in response as respondents were permitted to select more than one answer. Responses did not differ significantly (*p* = 0.62) between Faculty and students.

### Attitudes to spirituality in illness

The first part of this domain explored respondents’ attitudes to the relationship between spirituality and health status, and demonstrated a significantly wider range of opinions than those expressed above around more traditional components of health care. While 60 % of respondents overall felt that spiritual health contributes to physical well-being, almost a quarter felt it had a neutral effect and more than 15 % considered that it had no effect. Students tended to express more polarized views on the subject than Faculty (*p* = 0.005 for trends). Interestingly there was also a trend towards stronger support amongst Years 3–5 students than amongst Years 1–2 students (*p* = 0.03 for trend).

**Fig. 3:**
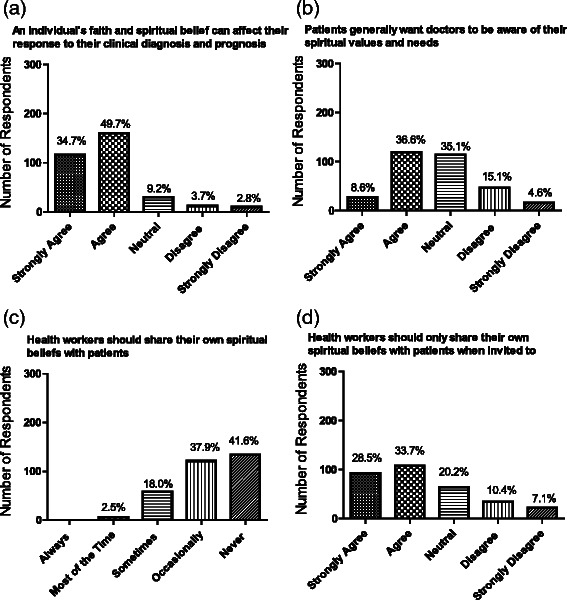
Respondents beliefs in respect of **a** how patients’ individual spirituality can influence clinical outcomes; **b** whether patients might wish health care staff to be aware their spiritual beliefs; **c** whether health care workers should share spiritual beliefs with patients overall, and **d** if they should do so only when invited. Data are given as absolute numbers and as percentages

Almost 90 % of respondents recognised that religious faith or personal spirituality was an important aspect of the lives of many patients. Similarly, 75 % of respondents felt that individual faith and spiritual beliefs can have an impact on response to diagnosis and prognosis in some patients (Fig. 3a, b). While overall there was general agreement that spiritual beliefs can impact care for some patients, students were generally more favourably disposed to the idea (87 % agreed or strongly agreed v 71 % of Faculty) with again fewer neutral opinions (7 % v 21 %; *p* = 0.02 for trend). There was less agreement in the perception of whether patients wished this to be communicated; over 40 % of respondents felt that patients generally wanted doctors to be aware of their religious values and needs, 20 % did not and ~33 % expressed a neutral view (Fig. 3c). Faculty were less inclined than students to support the idea that patients wished doctors to be aware of their spiritual values and needs (74 % Faculty were neutral/opposed vs 50 % of student respondents, I = 0.001 for trend). Similarly, Years 3–5 students tended to hold views more similar to Faculty and distinct from those students in Years 1–2 (for example 62 % of students in Years 1–2 agreed that patients wanted their spiritual values shared compared to 38 % of students in Years 3–5; *p* < 0.001 for trend).

In general, 80 % of respondents felt that healthcare workers should never or only occasionally share their own spiritual beliefs with patients and almost 67 % agreed that healthcare workers should only share such beliefs when invited to do so by patients (Fig. 3d). Students and Faculty expressed similar views. There was little consensus concerning the role of the doctor specifically in providing spiritual care. Overall, 45 % of respondents agreed or strongly agreed that spiritual care should be left to chaplains, while 30 % disagreed and 25 % had no strong view. A similar range of opinions was evident between Years 1–2 and Years 3–5 students, and between students overall and Faculty.

### Attitudes to inclusion of spiritual care training for health care staff

The overwhelming majority (94 %) of respondents indicated that they had not received any formal training in providing spiritual care to patients. Of ~6 % who had received some training, all were medical students, most of whom had undertaken the Student Selected Component (SSC) ‘Wholeness in Healing’ [24] in 2nd year, the remainder gaining some relevant experience via an SSC in multicultural medicine in 1st Year. No member of Faculty who responded to the survey had delivered any formal training in spiritual care to medical students (the study authors contribute to Wholeness in Healing but did not themselves complete the survey).

**Fig. 4:**
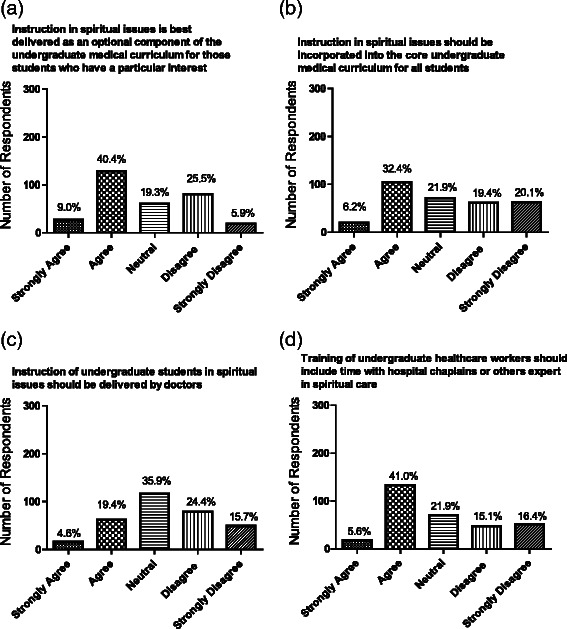
Respondent views on the place of spirituality in healthcare in the curriculum as **a** optional or **b** compulsory; and on who should be involved in providing students with training in spirituality in healthcare including **c** doctors and **d** chaplains. Data expressed as absolute numbers and as percentages

There was no clearly defined view as to how, or indeed whether, spiritual issues in healthcare should be addressed. ~47 % respondents agreed that awareness of world religions and faith practices should be part of the undergraduate curriculum, but ~33 % disagreed, and ~20 % had no strong views. The remaining questions in this section give insight into views on how instruction in spirituality in healthcare could be delivered, addressing its place in the curriculum, who should deliver, and the design of modules and assessment. If it was to be delivered, responses indicated that there was little agreement as to whether it should be compulsory. ~50 % respondents agreed that it should be an optional component of the undergraduate curriculum for students with a special interest, while 30 % disagreed (Fig. 4a); students tended to be more in favour of optional teaching than Faculty (51 % v 40 %; *p* = 0.056). Amongst students specifically, optional teaching was more strongly favoured (*p* = 0.03) by students in Years 3–5 than Years1-2; more experienced students were more opposed to compulsory teaching in this area (*p* = 0.02) compared to their less experienced colleagues. Overall, 40 % respondents felt such teaching should be incorporated into the core curriculum as a compulsory part of undergraduate medical student education, while an almost equal number disagreed (Fig. 4b); responses were similar between students and Faculty.

**Fig. 5:**
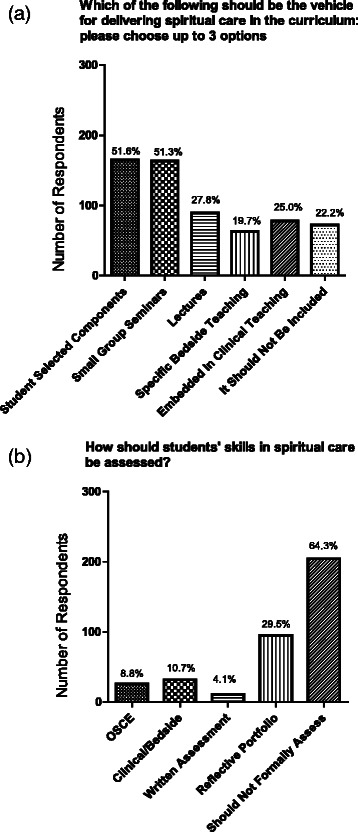
Respondent views regarding **a** vehicles for delivering teaching in spirituality for health care workers: **b** assessment of spirituality training in the undergraduate curriculum. Data are given as absolute numbers and percentage of responses

There was no consensus on whether doctors should deliver the training (~33 % neutral, 40 % against) (Fig. 4c). Students tended to be more supportive of doctors delivering the training than Faculty were to deliver (27 % students in favour v 8 % Faculty; *p* = 0.01 for overall trend) although the majority of students were still opposed to a role for Faculty in content delivery. ~50 % respondents agreed that training of undergraduate healthcare workers should include time with hospital chaplains or other experts in spiritual care (Fig. 4d). ~22 % respondents did not feel that there should be any spiritual care training provided in the curriculum. Of those who did endorse its use, student selected components and small group seminars were the most favoured modes of curriculum delivery. Less than 25 % supported embedding such teaching in a clinical setting (Fig. 5a).

67 % respondents were of the opinion that formal assessment should not be undertaken. This view was even more marked among Years 3–5 medical students (74 % against) compared to those in Years 1–2 (59 % against; *p* = 0.016 for trend). Of those supportive of formal assessment, the use of reflective portfolios was favoured (Fig. 5b).

### Thematic analyses of free text comments

Recurring viewpoints and themes were identified from the free text comments and are presented in categories below, with examples of comments in italics. Overall 91 of the 351 respondents (26 %) added free text comments, of which 88 expressed an analysable opinion on some aspect of the subject, and were included in the analysis.

### Respect for other views is the main issue

Respondents identified respect for patient views and beliefs as the key attribute for students but did not suggest any engagement with the individual in spiritual matters. Text comments expressing this belief were generally concise, such as:

*‘Respect....key principle!’*

Ideas were slightly more developed by other respondents but shared a common thread:

*‘The patient’s wishes with respect to spiritual care should be addressed. It is the responsibility of the medical and nursing team to facilitate access for the patient to whatever (or no) spiritual care they desire, but the medical and nursing staff should not be responsible for delivering that care. … It is important that medical students understand the impact of religious belief on people’s lifestyle or approach to death or treatment choices (e.g. around blood transfusion/transplantation) and can convey that they will respect that, and that they will help facilitate access to chaplains/other spiritual advisers for patient and family - but that decisions around input from religious communities remains entirely with the family and that the doctor retains the same respect for the patient and their family regardless of their decision re faith/spirituality’.*

### Spiritual aspects related to patient beliefs should be taught in the curriculum

Several respondents indicated that those aspects of spirituality which may impact on patient care should be included in the curriculum (for example knowledge about certain views on death, dying, cultural issues). However these respondents did not advocate direct medical involvement in spiritual issues and felt that all aspects of spiritual care should be dealt with by others. These respondents recommend that doctors are formally taught about and therefore have some understanding of the cultural and spiritual issues important to patients, but are not in any way engaged with them. Clearly these views overlap somewhat with the first group above, but in this case respondents actively endorsed courses to deliver the aim. Many comments in this vane were quite straightforward such as:

*‘doctors only need to know what religious practices could affect treatments or medications’.*

*‘I think this would be a worthwhile inclusion in the curriculum so that students gain an understanding of the way spiritually affects individuals and can potentially affect their health….an awareness is enough’.*

*‘I feel students need to know and understand different faiths and their beliefs related to their medical care - but should be neutral when it comes to ‘spirituality”.*

### A wider view of spirituality should be taught in the undergraduate curriculum

Responses dealing with the place of spirituality in healthcare in general, and specifically in the medical curriculum, could be divided into those strongly against any involvement, those strongly in favour of full inclusion, and those in favour of more limited inclusion such as in the previous section. An almost equal number of positive and negative responses were recorded. In this section the more positive responses are discussed. Some of the positive comments, often including justifications for inclusion of spirituality in a broad sense in the curriculum, are reproduced below:

*‘If spirituality is important to the patient then it is important in treatment of the patient’*.

*‘As healthcare professionals, doctors concern themselves with all aspects of a person’s life that can impact on their health and well-being. Regardless of your own personal beliefs, it is clear that to nearly all human beings there is acceptance or awareness of some level of spiritual reality in their life….. that actually tends to be most poignant in times of threat to health; doctors should certainly be more aware of the spirituality of the human person and have some ability to facilitate the engagement of this in the patient’s care.*

*‘…think since such an emphasis is put on “Holistic care” it only makes sense that medical students are taught about spirituality and how to handle these situations’.*

*‘I have heard examples of doctors sharing their faith with other patients with positive outcomes. ….I also believe that being ignorant of a patient’s beliefs or faith could result in flaws appearing within the doctor-patient relationship, increased problems when dealing with the family and ultimately it seems disrespectful’.*

Interestingly, some students responding to the survey noted that their own experiences in learning about spirituality in healthcare led them to espouse its inclusion in the curriculum.

*‘ I learnt a lot doing the spirituality SSC and feel that spiritual care should be included in the medical curriculum but spiritual care needs to be delivered by various people and is only enhanced by doctor’s participation’.*

### Spirituality should not be taught in the undergraduate curriculum

There was an almost equal number of clearly negative views on the place of spirituality in healthcare recorded in the free text comments of the questionnaire. Many arguments were marshalled against its inclusion with two frequently cited. The first was that matters of spirituality do not fit the biophysical medical model of disease, and are not ‘evidence-based’, and so should not be included.

*‘if we start including spirituality into a medical degree where the “evidence based” is emphasised, we shall also include homeopathy, herbal and voodoo medicine’.*

*‘having to provide spiritual counselling as a doctor diminishes the clinical scientific focus of the profession, as it is EVIDENCE BASED, and religion is not’.*

The second main argument is that doctors are not trained in spiritual matters and should leave all aspects of this to experts such as chaplains.

*‘If you want to talk about religion you go to a priest or whatever, if you want to talk about health you go to a doctor’.*

*‘The reason the hospitals have chaplains is to provide spiritual care to patients; it is the Doctor’s job to ensure the patient recovers physically, psychologically and emotionally. If they have spiritual or faith issues a chaplain is much better placed to address these’.*

Some of the negative responses came from respondents professing to be atheists, and some from those citing religious tensions in Northern Ireland as a reason not to address these issues. Interestingly some respondents identified themselves as Christians but felt unable to support spirituality in the curriculum due to perceived conflicts with GMC guidelines on sharing faith.

### A need for guidance

A separate theme coming through from the comments was the need for guidance for students in the difficult area of spirituality and interactions with patients. Comments were noted from both students and Faculty.

*‘There have been situations in hospitals where patients have referred to God/a higher power and I have not felt prepared by the medical curriculum, thus far, to respond most appropriately to this….. These are areas of grey, it seems, and guidance would be appreciated’.*

*‘I think it is important, as a Christian, to give some sort of formal teaching to all medical students about how to deal with tricky situations where you are unsure whether it is appropriate to share your faith or how you can assist patients if they ask you about spiritual things. . I think it’s also important to be aware, for all students, to understand the views of main religious groups that may affect the care you give them e.g. how they view death, burial, foods that can be eaten’.*

### Insights into medical education and spirituality

The final group of responses dealt with how spirituality could or should be dealt with in the curriculum. Respondents took the opportunity to expand on their views as expressed in domain 3. No respondent who commented felt that formal assessment should be included in any course, and several specifically recommended against it. In terms of the structure of any teaching, three recommended student selected components (optional) while two proposed core teaching including lectures. These comments were mainly factual statements of opinion and so examples are not included for reasons of brevity.

## Discussion

This study is novel in several respects. To our knowledge, it appears to be the first systematic report of views on spirituality in healthcare of medical students and Faculty in Northern Ireland, and possibly within UK, since most published studies report the North American experience. Direct comparison of the views of medical students and Faculty using the same instrument simultaneously has not been performed previously. Finally, assessment of student achievement of the learning outcomes of teaching of spirituality in healthcare is difficult and there is a paucity of published literature.

### Provision of spiritual care

There was clear support in our study for inclusion of physical, social and psychological aspects as components of patient care. Spiritual care was less convincingly acknowledged: while Faculty and students recognise that many patients consider spirituality is important and may influence their responses to illness, they as (future) healthcare workers are less convinced of its importance. This may be because fewer Faculty, and to an extent medical students, believe in a higher authority/God, compared to the general population of Northern Ireland (>83 %, which incidentally is higher than in other parts of UK, [28]). Similar trends have been identified in studies examining the views of medical students and Faculty relative to those of patients in USA [9, 29].

The majority of our respondents acknowledged that spiritual interventions should be available to patients, with counselling, access to a chaplain and /or faith representative the most commonly accepted interventions. Such interventions do not require direct participation of doctors, perhaps reflecting unwillingness to engage in delivery of spiritual care despite acknowledging its importance for some patients. Indeed most felt that doctors should rarely share their own beliefs with patients, and if so, only when initiated by the patient, preferring that delivery of spiritual care should be delegated to chaplains.

Overall, there is disconnection between theoretical understanding and acknowledgement of the role of spirituality, and its practical implementation into health care management. One possible explanation is that less than half of respondents perceived that patients actually wanted doctors to be aware of their personal beliefs and spirituality. However, studies conducted in USA in which patients were directly asked this question, reported that most patients in fact do [11]. Lack of time to undertake spiritual care, inadequate training, and difficulties in identifying those patients wishing to discuss spiritual issues may also impact doctors’ engagement [26, 30]. GMC guidance [31, 32] on the doctor/patient interface and sharing of religious faith with patients may also have struck a more cautionary note and deterred doctors from fuller engagement.

### Spirituality in the medical curriculum

The majority of respondents agreed that spiritual issues should be addressed somewhere in the undergraduate curriculum, but they were almost equally divided in regard to preference for compulsory teaching for all students and use of self-selected modules for those particularly interested. The current balance of our own course with a small amount of compulsory material and more in-depth optional teaching fits reasonably well with these stated views. Some respondents felt that teaching should be limited to imparting a basic working knowledge of major world religions and patient spirituality, without discussion of practical or care-based interventions. There are several descriptions in the literature of the content of medical courses addressing spiritual issues [18, 19] but little comment regarding what medical students themselves think should be included [26].

One survey of American medical students [29] felt such teaching should be provided in the earlier part of the course and that a lecture or seminar based approach would be most beneficial. The preference of our own Faculty and students for small group seminars is compatible with prominent use of such methods in many published curricula [19, 33, 34]. Puchalski and colleagues [35] generated recommendations based on identification of elements common to many American medical curricula, such as inclusion of appropriate literature, communication, spiritual assessment during history taking, breaking bad news, and knowledge of cultural and religious traditions; often the teaching method was inferred in such curricula, but not explicitly stated. Subsequently, they [36] formulated learning outcomes, which could potentially be mapped to teaching methods and assessment. A similar survey of teaching of spiritual care in UK medical schools [25] reported on content, aims and objectives of the small number of such courses currently delivered, but not on teaching methods although both classroom and clinic based approaches can be inferred.

Assessment of student learning of such issues receives very little attention in the literature, either in USA [20, 34] or UK [25, 26]. Fortin’s study [20] refers to use of student surveys or ‘self-reported changes in attitudes or knowledge’ to assess impact of such teaching, while direct observation of spiritual history taking skills is noted in one of the curricula described by Randall [25]. In the current survey, most respondents expressed the view that there should not be formal assessment; those who did support assessment favoured use of reflective portfolios. A reflection based assessment seems particularly well suited to this theme since it gives the option of both formative and summative components, as well as personal development and self-reflection all within the same tool [37]. Experience of inclusion of reflective portfolios within the assessment strategy of the ‘Wholeness of Healing’ SSC at QUB has been favourable [27].

Overall, the impression was that medical students were more favourably disposed towards the role of spirituality in health and disease than Faculty, though there was a trend towards more polarised student viewpoints. Direct comparison of Faculty and students’ views is largely lacking from the literature. Fortin’s review [20] stated that most US medical schools included student feedback in terms of satisfaction surveys, but gave no indication as to the feedback actually received. Most students undertaking a course of spirituality and healthcare in one American medical school [33] reported that completing the course would lead them to treat patients differently, but did not report their actual views on the specific content or delivery of the course. While overall, the attitudes amongst our own students were fairly similar, those in the earlier years of the course tended to be more favourably disposed to a relationship between spiritual and physical health and were more likely to recognise that many patients value spiritual care and wished their carers to be aware of their personal spirituality. That students moved towards the views of their teachers as they progressed in the course could have several explanations; a gradual maturing of opinion after exposure to real life clinical medicine could be relevant, and would account for the change from more polarised views earlier in the course. Indeed in a study conducted in USA, Sandor and colleagues [38] identified a reduction in ‘dogmatic perceptions’ of medical students and increased ‘spiritual maturity’ over time. A more provocative explanation is that the attitudes and opinions of the teachers consciously or subconsciously influenced the thinking of the students.

### Limitations and future studies

Although the majority of students did not respond, ~50 % Faculty and ~25 % of students did, with good representation from all year groups. The response is therefore likely to be representative and sufficient to allow meaningful differences in the scores for each question to be discriminated, based on power calculations. This does not guarantee absence of bias; for example those with particularly positive or negative attitudes toward spirituality may have been more inclined to reply to ensure their views were noted.

The questionnaire provides only a snapshot of opinion at a certain time; student views may change as they progress through the course. Future longitudional studies should seek to identify determinants of temporal changes in student opinions. Furthermore the questionnaire reflects the views of students at Queen’s University Medical School who are primarily, although not exclusively, from Northern Ireland. In addition, most of the students were school leavers in contrast to North American Medical Schools, from which most of the published literature is derived and medical study is normally restricted to graduate entrants. Several respondents to our study commented on the perceived attitudes of patients towards spiritual issues; future studies could usefully address the views of patients in Northern Ireland for comparison with the respondents in the current study. Finally assessment of student achievement of the learning outcomes of spirituality teaching remains a poorly researched area. A comparison of methods employed by various medical schools would be useful.

## Conclusions

There was general agreement in our study that all four components of whole person medicine (physical, psychological, social and spiritual) are important determinants of health and of patient care. Most respondents agreed that doctors should be aware of how matters of spirituality impact on patient attitudes towards diagnosis, prognosis and treatment. Most respondents felt that doctors should be aware of spiritual interventions which may benefit patients, including counselling, access to chaplains, and support from members of the patient’s faith community, but that doctors themselves should not provide spiritual care for patients.

Most respondents agreed that spiritual issues should be addressed in the undergraduate curriculum, but there was little consensus as to whether training should be compulsory or optional. Small group teaching, such as that afforded through the student selected component programme, was felt to be the most appropriate means for delivery. The majority felt that the theme should not be formally assessed in the curriculum, but if it was, reflective portfolios were considered the most appropriate assessment tool.

Given the mismatch between patient expectations and the reservation of some faculty and students regarding the importance of spiritual care, more needs to be done to encourage engagement of those who are reluctant to become involved in delivery of such care. Based on the current study, a number of recommendations are proposed. Generic themes such as the determinants and definitions of health, the influence of spirituality on patient attitudes and health related behaviours, the interaction between spirituality and disease, and awareness of the views and customs of the major World Faiths as they impact health related issues should be included in the core curriculum for all medical students, drawing on appropriate studies in the literature. In addition, all students should be aware of resources that can be mobilised to enhance the spiritual aspect of patient care and in particular how and when to refer to chaplaincy and allied services. The theoretical concepts would fit appropriately in the earlier years of the undergraduate course, supported by talks delivered by patients and clinicians sharing from personal experience of how their own spirituality has influenced their own health and care provided. It should be mandatory for all students in the latter years of their training to spend a day shadowing a chaplain on a hospital ward round to gain valuable insight into the impact of provision of such care for patients. The importance of self-care and support for students with mental health concerns has been highlighted in a recent GMC publication [39]. In response, at QUB for example we have recently introduced mandatory mental health awareness training for all students; such initiatives would provide a valuable opening to introduce the benefits of spiritual care, drawing on guidance from organisations such as the Royal College of Psychiatrists [40] and the Hospital Chaplains association (http://www.nihca.co.uk/).

In regard to assessment, use of OSCE stations utilising simulated patients/role-play should be explored; examples of scenarios piloted at Queen’s University Medical School are available ([26], online supplementary content) and could be made available as an online formative assessment resource for all students. In UK, all undergraduate and postgraduate medical students in training are required to maintain a reflective e-portfolio; inclusion of a reflective entry specifically on observed benefits of providing spiritual care could encourage greater engagement.

More detailed knowledge of spiritual care issues, including practical/clinical experience, should be available as an optional part of the course for example via student selected components for those students particularly interested in exploring this theme in greater depth. Amongst resources which may be mobilised in designing or revising undergraduate teaching on the spiritual aspect of healthcare, those we have found particularly useful are the draft learning objectives and suggestions for course design available via CETL (Barts and the London Medical School) [41] and (for a mainly Christian perspective) the training materials and courses on whole person medicine provided by Partnerships in International Medical Education [42].

## Additional file

Below is the link to the electronic supplementary material.

Additional file 1: **Survey of attitudes to whole person medicine and spirituality.**
