# Supplementary material for: How should teaching on whole person medicine, including spiritual issues, be delivered in the undergraduate medical curriculum in the United Kingdom?
Source: BMC Med Educ. 2015 Jun 2;15:96. doi: 10.1186/s12909-015-0378-2 (PMC4460734; doi:10.1186/s12909-015-0378-2)
Supplement: Supplementary file 1 — Survey of attitudes to whole person medicine and spirituality. [file 12909_2015_378_MOESM1_ESM.docx]

# Additional file 1 –SURVEY OF ATTITUDES TO WHOLE PERSON MEDICINE AND SPIRITUALITY

The purpose of this questionnaire is to ascertain the views of those involved in health care training and education on issues related to spirituality and whole person care. For this study we define spirituality as ‘personally held beliefs, values, and practices’ and spiritual care as ‘an awareness of and sensitivity to the spiritual values of patients, and the provision and mobilization of spiritual resources appropriate to the patient in their situation’. We are interested to know the attitudes of students and medical teachers to this subject, and how it may fit into the medical curriculum. The results from this survey will form part of a research project and degree dissertation undertaken at Queen’s University investigating attitudes towards whole person care.

**What we are asking you to do**

We are asking for a small part of your time to share your views with us by answering the following questions. This survey is confidential and anonymous and no personal identifiable data will be sought or stored. We would be very grateful if you could find some time to complete the survey. If you feel unable to respond to all the questions, please answer all questions you feel able to. You may withdraw from completing the questionnaire at any stage. Participation in this survey is entirely voluntary; please do not feel under any obligation to participate. Your participation or non-participation will not affect your career progression or any aspect of your undergraduate performance. If you have any queries about this project please contact the study supervisor. Thank you for your time.

# DEMOGRAPHICS

1. Please indicate if you are a:

QUB staff-clinical [ ] QUB staff-non clinical [ ]

Medical student-school leaver [ ] Medical student- graduate entry [ ]

2. Please indicate your age:

<20 years [ ] 21-25 years [ ] 26-30 years [ ] > 31 years [ ]

3. Please indicate your gender:

Male [ ] Female [ ]

4. Please indicate your country of birth:

N. Ireland [ ] ROI [ ] Great Britain [ ] Europe [ ] Rest of World [ ]

5. If you are a medical student please indicate your year of study:

First [ ] Second [ ] Third [ ] Fourth [ ] Final [ ]

# ATTITUDES TO WHOLE PERSON MEDICINE

Please indicate how important you think each subject is in the management of patients:

6. Physical treatment (drugs/surgery)

Very important [ ] important [ ] neutral [ ] little importance [ ] irrelevant [ ]

7. Social care

Very important [ ] important [ ] neutral [ ] little importance [ ] irrelevant [ ]

8. Psychological care

Very important [ ] important [ ] neutral [ ] little importance [ ] irrelevant [ ]

9. Spiritual care

Very important [ ] important [ ] neutral [ ] little importance [ ] irrelevant [ ]

10. Which of the following do you consider most important in the provision of spiritual care for patients? Please tick all that apply:

Counselling [ ] Sacred Music/Songs [ ] Religious rites/ceremonies [ ] Healing services [ ]

Access to a chaplain [ ] Access to sacred texts/readings [ ] Prayer / intercession [ ]

Contact with a member of patient’s own Faith Community [ ] None of these [ ]

Other [ ] Please detail

# ATTITUDES TO SPIRITUALITY IN ILLNESS

Please indicate how strongly you agree with each statement:

11. Spiritual health contributes to physical health

Strongly agree [ ] Agree [ ] Neutral [ ] Disagree [ ] Strongly disagree [ ]

12. Religious faith or personal spirituality is an important aspect of the lives of many patients

Strongly agree [ ] Agree [ ] Neutral [ ] Disagree [ ] Strongly disagree [ ]

13. Patients generally want doctors to be aware of their religious/spiritual values and needs

Strongly agree [ ] Agree [ ] Neutral [ ] Disagree [ ] Strongly disagree [ ]

14. Health workers should share their own spiritual beliefs with patients

never [ ] occasionally [ ] sometimes [ ] most of the time [ ] always [ ]

15. Health workers should only share their own spiritual beliefs with patients when invited

Strongly agree [ ] Agree [ ] Neutral [ ] Disagree [ ] Strongly disagree [ ]

16. Doctors should leave spiritual care to chaplains or others

Strongly agree [ ] Agree [ ] Neutral [ ] Disagree [ ] Strongly disagree [ ]

17. An individual’s faith and spiritual belief can affect their response to their clinical diagnosis and subsequent prognosis

Strongly agree [ ] Agree [ ] Neutral [ ] Disagree [ ] Strongly disagree [ ]

18. Sometimes patients recover for reasons which cannot be explained medically or scientifically

Strongly agree [ ] Agree [ ] Neutral [ ] Disagree [ ] Strongly disagree [ ]

**ATTITUDES TO SPIRITUAL CARE IN THE TRAINING OF HEALTH CARE STAFF**

19. Have you received any formal training in providing spiritual care to patients?

Not applicable [ ] No [ ] Yes [ ] if yes please detail

20. Have you delivered any formal training in providing spiritual care to patients?

Not applicable [ ] No [ ] Yes [ ] if yes please detail

Please indicate how strongly you agree with each statement:

21. Instruction regarding world religions and faith practices should be part of the medical undergraduate curriculum

Strongly agree [ ] Agree [ ] Neutral [ ] Disagree [ ] Strongly disagree [ ]

22. Instruction in spiritual issues is best delivered as an optional component of the undergraduate medical curriculum for those students who have a particular interest

Strongly agree [ ] Agree [ ] Neutral [ ] Disagree [ ] Strongly disagree [ ]

23. Instruction in spiritual issues should be incorporated into the core undergraduate medical curriculum for all students

Strongly agree [ ] Agree [ ] Neutral [ ] Disagree [ ] Strongly disagree [ ]

24. Instruction of undergraduate students in spiritual issues should be delivered by doctors

Strongly agree [ ] Agree [ ] Neutral [ ] Disagree [ ] Strongly disagree [ ]

25. Training of undergraduate healthcare workers should include time with hospital chaplains or others expert in spiritual care

Strongly agree [ ] Agree [ ] Neutral [ ] Disagree [ ] Strongly disagree [ ]

26. Which of the following should be the vehicle for delivering spiritual care in the curriculum (please tick up to 3 options):

Student selected components [ ] Small group seminars [ ] Lectures [ ]

Specific bedside teaching [ ] Embedded in clinical teaching [ ]

It should not be included [ ]

27. How should students’ skills in spiritual care be assessed

OSCE [ ] Clinical/bedside [ ] Written assessment [ ] Reflective portfolio [ ]

It should not be formally assessed [ ]

28. Do you believe in God or a higher power/authority?

Yes [ ] No [ ] Do not wish to disclose [ ]

Please write below any comments you would like to add on this subject**:**
